# Barriers and facilitators to housing and healthcare services for people experiencing homelessness with concurrent brain injury, mental health and substance use disorders: a qualitative study

**DOI:** 10.3389/fpubh.2025.1643689

**Published:** 2025-08-19

**Authors:** Grace C. Warren, Cole J. Kennedy, Nicholas Gavas, Julia Schmidt, Erica Woodin, Janelle Breese Biagioni, Mauricio A. Garcia-Barrera

**Affiliations:** ^1^Department of Psychology, University of Victoria, Victoria, BC, Canada; ^2^Institute on Aging & Lifelong Health, University of Victoria, Victoria, BC, Canada; ^3^Canadian Institute for Substance Use Research, Victoria, BC, Canada; ^4^Rehabilitation Research Program, Centre for Aging SMART at Vancouver Coastal Health, Vancouver, BC, Canada; ^5^Department of Occupational Science and Occupational Therapy, University of British Columbia, Vancouver, BC, Canada; ^6^CGB Centre for Traumatic Life Losses, Victoria, BC, Canada

**Keywords:** acquired brain injury, homelessness, substance use, mental health, qualitative research, community-engaged research

## Abstract

**Background:**

Acquired brain injury (ABI) can significantly impact mental health, vulnerability to addictions, and housing stability, yet the intersection of these challenges is understudied. Individuals living with ABI are disproportionately represented among populations experiencing homelessness and have a high prevalence of concurrent mental health and substance use (MHSU) disorders, leading to poorer health outcomes and lower quality of life. The objective of this study was to identify barriers and facilitators to housing and healthcare services for people experiencing homelessness with ABI and concurrent MHSU disorders.

**Methods:**

Data were collected during a one-day workshop as part of the *British Columbia Consensus for Brain Injury, Mental Health and Addiction* project. Semi-structured focus groups involving ABI survivors, service providers, and community stakeholders explored barriers, facilitators, and recommendations for service improvements. Using manifest content analysis, data were analyzed in accordance with a well-validated conceptual framework for healthcare access.

**Results:**

A total of 163 stakeholders (*M* = 46.40, *SD* = 13.80, 72% female) including 74 with lived experience of ABI and/or homelessness, participated in the focus groups. Manifest content analysis revealed five barriers and five facilitators: Barriers included (1) *Stigma*, (2) *Insufficient Investment*, (3) *Siloed Systems*, (4) *Generalized Approaches to Housing*, and (5) *Policies that do not Support Complex Needs*, while facilitators included (1) *Increasing Discourse on the Intersections of ABI, MHSU, and Homelessness*, (2) *Government Commitment to Systemic Change*, (3) *Collaboration Across Organizations*, (4) *Community-Based Services*, and (5) *Supportive Housing Models*.

**Conclusions:**

These findings highlight gaps in existing policies and services while identifying effective approaches to supporting individuals experiencing these intersections. Efforts to address barriers and leverage existing facilitators may support the development of accessible services that address unmet health and housing needs among people experiencing homelessness with concurrent ABI and MHSU conditions.

## 1 Introduction

Acquired brain injury (ABI), mental health and substance use (MHSU) disorders, and homelessness are pervasive and intersectional global public health crises (1, 2). ABI, consisting of both traumatic brain injury (TBI) and non-traumatic (nTBI) subtypes, is a chronic and life-altering condition resulting from damage to brain tissue and functioning. TBI is the leading cause of death and disability worldwide (1), with lifetime prevalence estimates ranging from 19 to 29% (3). While every ABI is unique, it is common for survivors to experience changes to their cognitive, physical, behavioral, and emotional functioning (4, 5). Anxiety disorders and mood disorders are commonly diagnosed in the first 12 months post-injury, with prevalence rates of 44.1% and 42.2%, respectively, among TBI survivors (4). These mental health challenges are often accompanied by feelings of grief and loss as survivors and their families navigate changes to self-concept, interpersonal relationships, and daily life post-injury (6). Additionally, TBI research suggests 10%−20% of survivors will develop a substance use disorder post-injury, even with no pre-injury history of substance dependence (7). Often, post-injury substance use behaviors are underpinned by cognitive deficits in risk evaluation or inhibition (7–9) and physical or psychological pain (10), for which substances serve as a coping mechanism (11). Post-injury circumstances can make it very difficult for ABI survivors to maintain relationships, seek or return to employment (12, 13), complete activities of daily living, navigate systems, and access support services (14). While there are numerous circumstances that can lead to experiences of homelessness, the pervasive impacts of ABI on an individual's interpersonal, emotional, and vocational functioning may explain in part why ABI survivors are disproportionately represented among populations experiencing homelessness (15, 16).

Homelessness is a leading public health issue, affecting over 100 million people globally (17). People experiencing homelessness (PEH), including people without accommodation, people living in temporary or crisis accommodation (i.e., emergency shelters), and people living in severely inadequate and insecure accommodation (18), face a wide range of health inequities that contribute to higher rates of morbidity and mortality among this population (19–22). In Canada, it is estimated that 76.2% of the approximately 235,000 PEH have one or more mental health disorders (22, 23). Among the mental health disorders they experience (e.g., schizophrenia, major depression, bipolar disorder, personality disorders), the most common are alcohol and drug use disorders, with prevalence rates 36.7% and 21.7%, respectively (22). Furthermore, meta-analytic data reveals that the prevalence of any severity of TBI among homeless and marginally housed populations is 53.1%, a rate between 2.5 and 4-times higher than that of the general population (16). Among PEH with ABI, 22.5% have survived a moderate to severe TBI, exceeding the general population prevalence by almost 10 times (16). Importantly, meta-analytic data indicates that the majority (51%−92%) of these individuals obtained a TBI before becoming unhoused, and that a history of TBI is associated with homelessness at a younger age as well as poorer health outcomes (15, 24). While ABI is a significant risk factor for experiencing homelessness, the living conditions associated with homelessness have also been shown to increase the likelihood of sustaining an ABI.

In these conditions, individuals are vulnerable to violent attacks, falls, and engagement in risky behaviors that all have the potential to result in TBI (25). Rising global drug toxicity and use in PEH also contributes to an increased prevalence of nTBI subtypes including hypoxic/anoxic brain injury due to opioid overdose (26–29). According to reports from the British Columbia (BC) Coroners Service, drug toxicity has consistently been the leading cause of death among PEH in BC since 2016, accounting for 86% of the 458 reported deaths in 2023 (30). Notably, this data does not reflect the high prevalence of individuals who survive an overdose event and, as a result, may be living with ABI (27–29, 31). Taken together, these findings suggest it is highly plausible that a significant majority of PEH have sustained an ABI, whether a TBI or nTBI, at some point in their lives. Action is needed to address this pervasive health crisis.

While there remains a significant need for mental and physical healthcare services among this population, PEH report several barriers to receiving care. For instance, from their ethnographic observations in Dublin, Ireland, O'Carroll and Wainwright (32) established that both administrative (e.g., applications for medical care, appointments, rules of service, management of addiction in hospital) and attitudinal (e.g., stigma) factors posed significant barriers to accessing healthcare services for PEH. Additionally, PEH reported internalized barriers to care including the presumption of poor treatment or discrimination, fear, embarrassment, self-blame, and hopelessness. These barriers to care are especially pronounced for PEH with ABI (33). When experienced together, ABI, MHSU disorders, and homelessness create complex and unique needs that are often unmet by current housing and healthcare approaches (14, 34). For example, the siloed nature of healthcare, housing, mental health, and addiction services places the burden of system navigation on survivors of ABI and PEH, who may experience cognitive impairments post-injury (e.g., executive function deficits, memory impairments, emotional dysregulation) that make it challenging to complete lengthy or complex tasks required to engage in services (e.g., seeking medical and housing opportunities, completing paperwork, and managing finances) (8, 12, 14). Similarly, concurrent MHSU challenges can exclude individuals from housing eligibility, while also compounding ABI-related symptoms and contributing to the complexity of needs (35). The current North American housing crisis significantly limits opportunities for affordable housing (36), particularly among those facing financial limitations due to post-injury circumstances. Within available housing, individuals with concurrent ABI and MHSU challenges experiencing homelessness may face further disadvantages due to stigma and discriminatory practices from housing providers and landlords (37). The same is true within healthcare service provision, where many PEH and survivors of ABI report experiencing stigma, contributing to increasingly disparate health outcomes (14, 32).

While it is crucial to understand barriers to care, it is equally important to recognize and leverage effective approaches. Building upon existing models and practices that have proven successful in supporting the unique needs of this population is a vital step toward promoting equitable care. Access to safe, secure, and appropriate housing is not only a basic human right, but also a critical component of recovery and prevention for individuals navigating ABI, MHSU, and homelessness (38). Among PEH living with a disabling condition (i.e., recognized as having limitations in daily activities, an inability to work or live independently, or the presence of HIV infection), Housing First models that integrate individualized MHSU support have been shown to reduce homelessness by 88%, promote housing stability by 41%, and improve community integration and quality of life overall (39). Such integrative approaches have been endorsed by previous research as particularly effective in supporting survivors of ABI with concurrent MHSU support needs (37). Additionally, improving housing stability among this population can minimize exposure to preventable TBI (25), reduce substance use behaviors, and improve social connection and mental health (37). These findings speak to the critical need for adequate housing as a foundation to addressing concurrent challenges among PEH with ABI and MHSU conditions. Moreover, it is essential to understand which factors facilitate or impede access to these necessary supports to recommend evidence-informed changes that maximize benefit to this underserved population.

The existing literature provides several meaningful contributions to inform our understanding of the intersections of ABI, MHSU, and homelessness. However, gaps remain in our knowledge of how these factors, especially when experienced together, affect individuals navigating healthcare and housing systems. Given the significant overlap among these challenges and the disproportionately high prevalence of ABI and MHSU among PEH (16, 40–43), it is essential to approach these topics in an integrated manner to ensure the unique needs of this population are accurately reflected in the ideas and recommendations for developing accessible services. While some research has explored reciprocal relationships between ABI, MHSU, and homelessness, few studies consider their combined effects (2, 4, 11, 16, 22, 34, 37, 42). Moreover, existing qualitative work often relies on individual interviews, limiting opportunities for stakeholder dialogue and collaboration (37).

Therefore, this study seeks to address these gaps by adopting a participatory action research (PAR) approach (44) to examine how concurrent ABI, MHSU, and homelessness shape access to healthcare and housing in the Canadian context. This approach aligns with recommendations to collaborate across sectors to develop innovative strategies for care (37). Involving individuals with lived experience and diverse community partners as knowledge holders offers a unique advantage in the co-creation of actionable and contextually relevant recommendations that will serve to address fragmentation both in research and service structures. Importantly, co-designing housing and healthcare approaches with individuals who have lived experience can support best practices that are informed by those receiving care, rather than defined solely by system-centric approaches. Additionally, this study responds directly to stakeholder-driven priorities for research addressing the intersections of ABI and MHSU (45), where the number one rank-ordered priority for research is to understand how experiences of ABI and MHSU differ between PEH and those with stable housing. To better understand the complex interactions between individual, systemic, and structural barriers and facilitators, this study draws upon a well-validated conceptual framework of access to healthcare by Levesque and colleagues (46, 47) as a guiding lens for qualitative content analysis. This framework offers a structured approach to identifying how service delivery corresponds with the needs of different populations to shape access to care–a critical roadmap to the present study.

## 2 Methods

### 2.1 Study design

The present study was conducted as part of the *BC Consensus for Brain Injury, Mental Health and Addiction* project (subsequently referred to as the BC Consensus on Brain Injury), a 3-year research initiative to understand the priorities and solutions to best serve people experiencing the intersections of brain injury, mental health, and addictions in BC (14, 33, 45). This study uses data collected in the third and final year of the project, which had an emphasis on understanding these factors as they relate to housing and homelessness. The present study employed a PAR approach, which emphasizes empowerment, collaboration, and creative problem-solving in the creation of actionable solutions (44). Importantly, PAR aims to provide meaningful and relevant research findings by involving community knowledge systems and individuals with lived experience throughout the research process and translating these findings into informed changes that directly support participants once research is completed (44). Additionally, elements of appreciative inquiry (48) were embedded in this study's framework, which oriented conversations toward solution-focused and strengths-based recommendations. A qualitative methodology was chosen to best capture the phenomenology of intersecting ABI, MHSU, and homelessness, resulting in data that reflect the lived experiences informing many participants' perspectives and recommendations. Given that many of the participants had lived experience, the use of manifest content analysis (49) allowed us to reflect their insights in the priorities and solutions identified. Findings are reported according to the COnsolidated criteria for REporting Qualitative research (COREQ) Checklist. Ethics approval for this study was obtained by the Research Ethics Board of the University of Victoria (#22-0614) and the University of British Columbia (#H22-03403).

### 2.2 Participants

Participants were recruited through outreach efforts targeting organizations and stakeholders across BC including brain injury support services, healthcare providers, health authorities, government representatives, and other community partners. Recruitment messages were distributed in person and via email, asking these organizations to refer individuals whose experiences or professional roles intersect with ABI, MHSU, housing, and homelessness. This approach sought to engage a diverse range of participants, such as individuals with lived experience (e.g., people navigating brain injury and/or homelessness and their family members), service providers, researchers, policymakers, healthcare workers, and representatives from brain injury organizations. A strong emphasis was placed on ensuring representation from equity-deserving groups, including Indigenous peoples, individuals with disabilities, and members of the 2SLGBTQIA+ community. These groups are disproportionately affected by ABI, MHSU, and homelessness (50–54), and the meaningful inclusion of their voices and perspectives are essential to shaping recommendations that are culturally relevant, inclusive, and accessible.

Participation in the one-day event (hereafter referred to as the Consensus Building Day) required informed consent, which was obtained through an online survey. The survey also gathered demographic details such as age, ethnicity, gender, and sex, alongside participants' self-identified roles in relation to the research (e.g., researcher, service provider, individual with lived experience). Participants could select multiple roles if applicable, including the opportunity to disclose whether they identified as a survivor of brain injury or a person with lived experience of homelessness. In accordance with the BC Center for Disease Control Peer Payment Standards, participants with lived experience were compensated for sharing their time and perspectives (55). All recruitment processes followed best practices in Equity, Diversity, and Inclusion in Research as detailed by the New Frontiers in Research Fund (NFRF) and the Canadian research Coordinating Committee (CRCC).

### 2.3 Procedure

Data were collected at the Consensus Building Day that took place on June 20, 2024, both in-person at the University of Victoria and simultaneously online (via Zoom). The process for the day was identical for both in-person and virtual participants. Participants were provided context for the goals and anticipated outcomes of the day to foster productive conversations. Before commencing group work, participants heard from a panel discussing the intersections of ABI, MHSU, and homelessness and the importance of understanding these health priorities collectively. Participants then joined their focus groups, which were assigned based on their self-reported role and connection to ABI, MHSU, and homelessness. For example, researchers aimed to have an individual with lived experience, a healthcare professional, a policy maker, and a caregiver represented among each group to diversify the opinions, experiences, and ideas shared. Focus group sizes ranged from 7 to 11 participants, with each group facilitated by a trained moderator who had previous experience working with vulnerable populations. All 16 table moderators attended a 1-hour training session prior to the event to ensure they were equipped with the skills and knowledge necessary to effectively manage personal biases, facilitate group dynamics, and allow all voices to be heard. Each table was also supported by a designated scribe, whose responsibilities were to record participants' ideas and quotes throughout the focused group work. The overall activities and presentations throughout the day were facilitated by a Certified Facilitator who was external to the research team. The data collection process and programming throughout the day followed a similar structure to the years prior, as outlined in previous publications (14, 33, 45).

### 2.4 Data collection

Group work began with a round table introduction where participants had the opportunity to share their connection to the research or self-identified role(s) (e.g., person with lived experience, healthcare worker, case manager). Table moderators were invited to share their connections to these topics to promote transparency. Participants were then guided through the first data collection portion of the day, where groups brainstormed barriers, facilitators, and recommendations to improve access to housing and healthcare services. Three separate open-ended questions were posed, and the process by which ideas were shared and recorded was identical for each question. The questions were as follows: *Keeping the emphasis on the role of housing in mind*, (1) *What are one or two things about mental health and addictions care for people with brain injury in BC that are working really well?* (2) W*hat are one or two things about mental health and addictions care for people with brain injury in BC that are not working very well?* and (3) *If you could change one thing about how brain injury is managed in BC, what would it be?* These questions were explored through table group conversations facilitated by the table moderators.

Participants were guided through the idea generation and data collection process, working with one question at a time. First, table moderators read the question out loud to participants and asked them to reflect individually on their ideas for a few minutes, writing down notes if desired. Table moderators then asked participants to share what they believed to be the 1–3 most important ideas from their list, reading their ideas to the group one at a time. All ideas were relayed back to the participants to ensure their thoughts were accurately represented before recording them. Once a list of all the participants' ideas had been created, table moderators worked with participants to discuss which concepts were similar and could be grouped together. Throughout this discussion process, the table moderators ensured group dynamics were balanced, and each participant had the opportunity to share their thoughts within the group dialogue. Finally, after grouping similar ideas, participants worked together to create a descriptive label for each category of ideas. These labels were then shared with the event facilitator and organizing committee. Although quotes were not a necessary component for data analysis within this research, they were recorded throughout the group discussions to reflect salient ideas and experiences shared by participants that ground the summarized data in tangible lived experiences.

### 2.5 Analytic strategy

**Figure 1:**
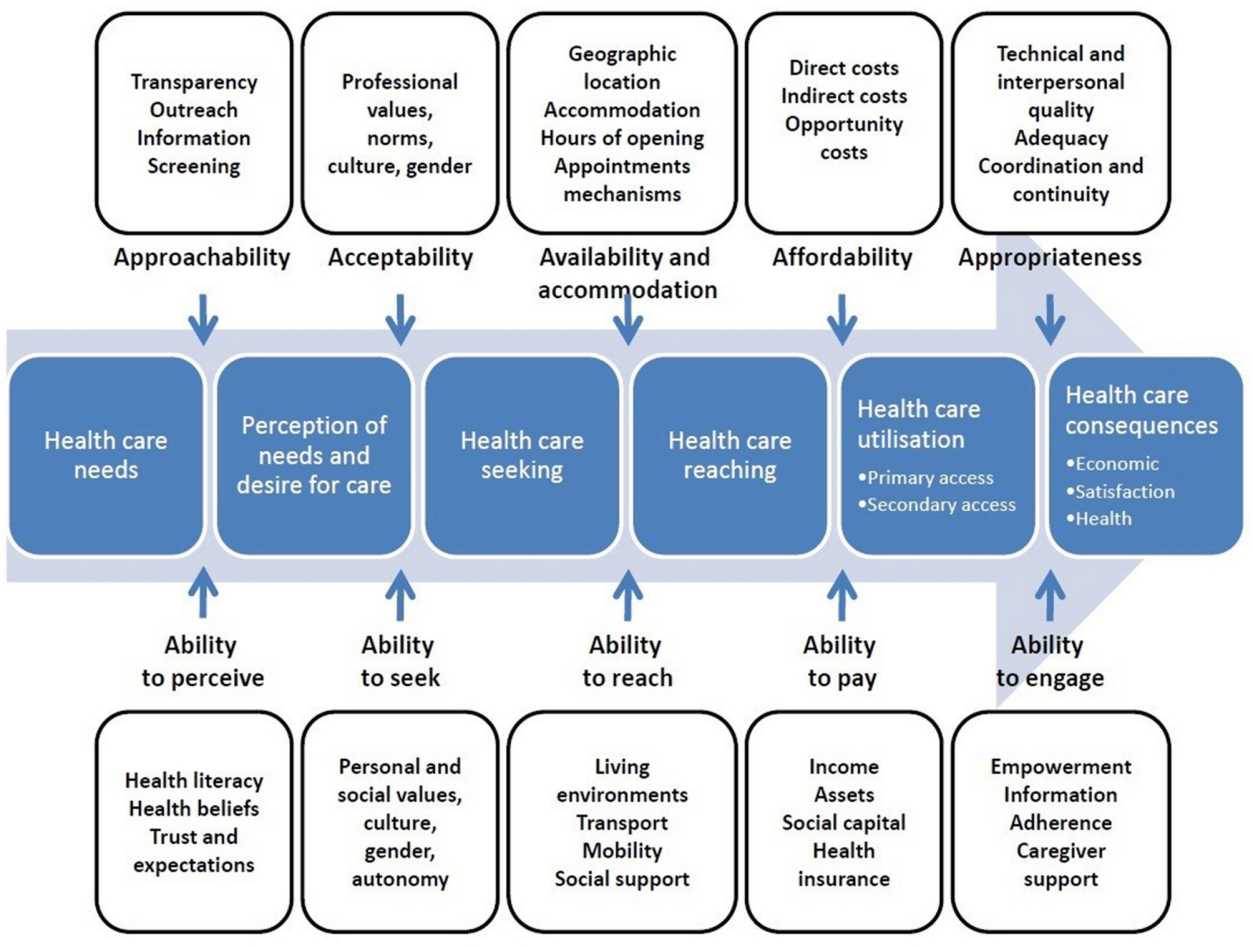
A conceptual framework of access to healthcare by Levesque et al. (46). This figure represents the dimensions of accessibility within a conceptual framework of access to healthcare. Reprinted from patient-centered access to health care: Conceptualising access at the interface of health systems and populations by Levesque et al. (46).

Manifest content analysis (49) was used to analyze the group-level summarized data. This approach served to reflect the wide range of ideas generated while retaining the surface-level content that was directly expressed by participants. This method also aligned closely with the study's overarching goal of reaching a consensus that encompasses many diverse ideas and perspectives, rather than exploring in-depth individual responses. Preliminary coding processes were grounded in a well-validated conceptual framework of access to healthcare (46, 47). The framework, illustrated in Figure 1, has five comprehensive categories that reflect the dimensions of accessibility from both the service provider and service user perspectives (46). In their original paper, Levesque and colleagues operationalized these dimensions as follows.

First, *Approachability & Ability to Perceive* reflect the visibility and transparency of services and information about how to access them, as well as an individual's health literacy, health beliefs, trust, and expectations that impact the likelihood of them identifying a health need and seeking services to support themselves. Second, *Acceptability & Ability to Seek* refer to the alignment between existing services and the societal or cultural norms that may influence an individual's willingness or ability to make use of them. For example, cultural and gender considerations in service delivery may impact the likelihood an individual will accept the care delivered, while sharing information about rights to care may promote an individual's autonomy and ability to seek services. Third, *Availability and Accommodation & Ability to Reach* represent the physical availability and accessibility of services (e.g., location, hours, physical accessibility, appointment mechanisms) as well as their capacity to provide care (e.g., presence of healthcare professionals, qualifications). Fourth, *Affordability & Ability to Pay* underscore the direct and indirect costs of engaging with services (e.g., financial costs, opportunity costs) and the resources needed to support this process (e.g., income, health insurance, assets). Lastly, *Appropriateness & Ability to Engage* reflect how well services fit patients' unique needs (e.g., technical and interpersonal quality, timeliness, coordination and continuity of care, assessment of health needs to provide appropriate support) and the patient's ability to participate in their care and treatment decisions.

While this model was originally designed to assess healthcare accessibility, it reflects many of the same mechanisms that relate to housing accessibility and was therefore deemed appropriate to use for the current study, which focuses on the barriers and facilitators to both healthcare and housing. Additional information about the model and its use in healthcare research can be found elsewhere (46, 47).

Prior to beginning data analysis for the present study, two independent coders GW (female, undergraduate student, researcher, experience with ABI population in community, research, and personal contexts) and NG (male, undergraduate student, researcher, experience with ABI population in research context) applied this framework to pilot data from Year One of the BC Consensus on Brain Injury project, which focused on the barriers and facilitators to healthcare in the context of overdose survival (14). Coders used the qualitative data analysis software NVivo (version 14.23.3) to categorize pilot data into the five dimensions of accessibility, meeting regularly to discuss the operationalization of codes as needed. Based on the strong inter-rater agreement (90.70%) from preliminary coding of the pilot data and the perceived alignment between the model's categories and identified data patterns, we decided the model was a suitable fit for understanding data from the present study. Coders immersed themselves in the data before analyzing its content. Next, they independently coded all barriers and facilitators using NVivo in accordance with a shared codebook, which outlined the operationalized dimensions of accessibility as described above. This preliminary coding process served to triage data into similar categories that are grounded in theory before exploring emergent categories that reflect the specific barriers and facilitators to housing and healthcare. Following preliminary coding, GW independently coded emergent categories, which were refined through discussions with supervisors CJK and MGB to ensure the outcomes of codes and categories from the initial analyses were representative, comprehensive, and mutually exclusive.

## 3 Results

### 3.1 Participant characteristics

**Figure 2:**
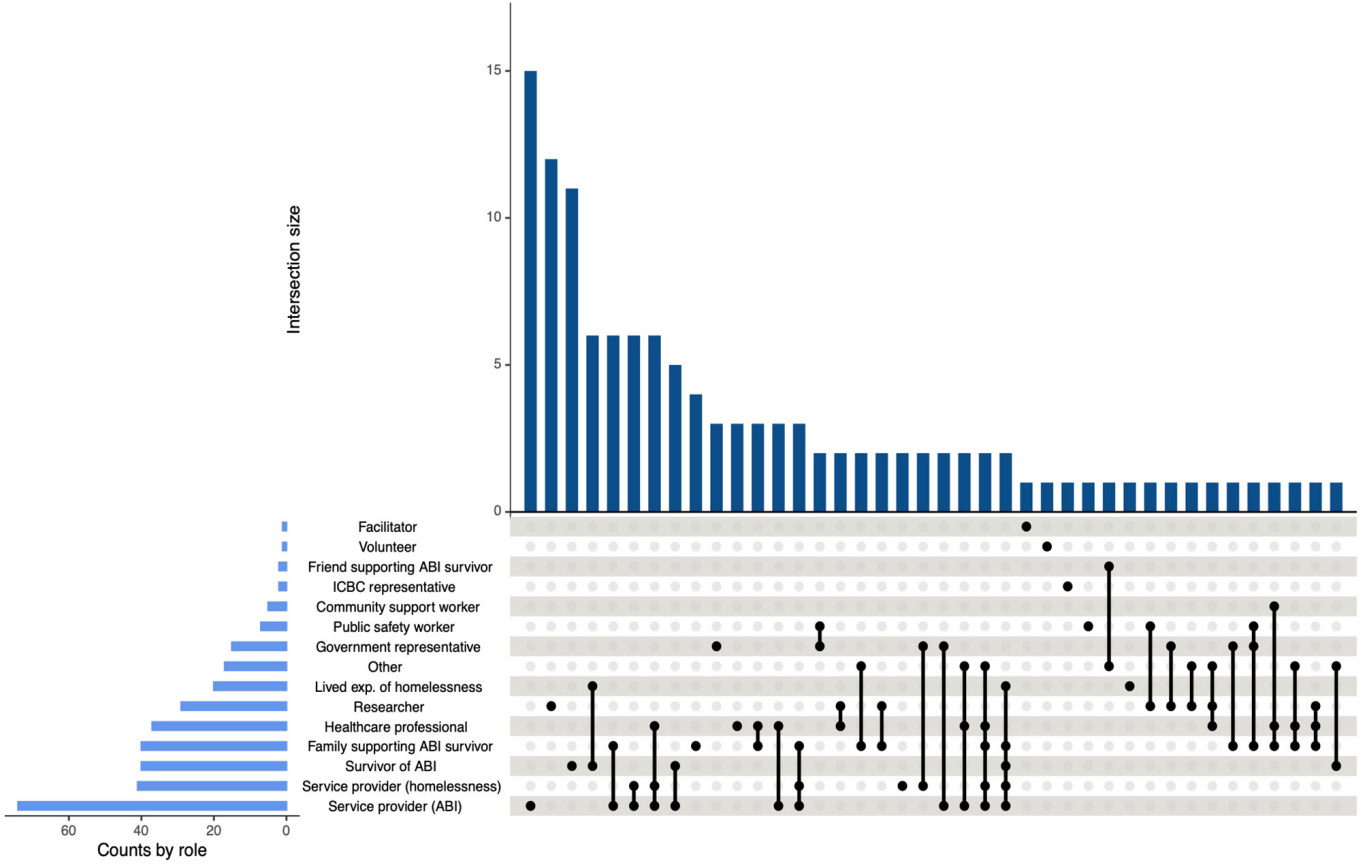
UpSet plot of intersecting self-identified roles among participants. Figure demonstrating the frequency of intersecting self-identified roles among participants. The total frequency of roles across all intersections is indicated by the horizontal bars next to each role title, while the frequency of each intersection is represented by the vertical bars above each grouping of roles. An individual dot next to a role title indicates a subgroup of participants who identified with that role and no additional roles.

In total, 163 individuals participated in the event. Of these, four opted not to share demographic data and were excluded from the demographic analyses. Participant ages ranged from 20 to 70 years (*M* = 46.40, *SD* = 13.80). The majority of participants identified their sex as female (*n* = 114, 72%) and their gender as a woman (*n* = 114, 72%). While most participants (*n* = 123, 77%) identified as heterosexual, there was some representation from individuals of diverse sexualities (*n* = 21, 13%). Regarding racial and ethnic backgrounds, the majority of participants (*n* = 126, 79%) identified as White (e.g., of British, French, German, or North/South American European ancestry). Notably, there was representation from individuals who identified as Indigenous or of mixed-Indigenous ancestry (*n* = 18, 11%). Additional information about the sociodemographic characteristics of the sample is provided in Table 1. The most common self-identified roles among participants included service providers for ABI (*n* = 74, 47%) and service providers for PEH (*n* = 41, 26%), followed by survivors of ABI (*n* = 40, 25%) and family members supporting survivors of ABI (*n* = 40, 25%). Several participants reported dual or multiple roles, which were visualized using an UpSet plot and displayed in Figure 2. Notably, the most frequent intersection of self-identified roles was between individuals with lived experience of both ABI and homelessness.

**Table 1:** Sociodemographic characteristics of participants.

**Sample characteristics (*N* = 159)**	***n***	***%***
Age (*M, SD*)	46.40, 13.80	
**Sex**		
Female	114	71.70
Male	39	24.53
Prefer not to answer	6	3.77
**Gender identity**		
Woman^*^	114	71.70
Man^*^	40	25.16
Prefer not to answer	5	3.14
**Sexual orientation**		
Heterosexual	123	77.36
Homosexual	8	5.03
Bisexual	8	5.03
Pansexual	2	1.26
Queer	2	1.26
Asexual	1	0.63
Prefer not to answer	15	9.43
**Race or ethnic background**		
White (e.g., British, French, German, North or South American of European background)	126	79.24
Indigenous Person (e.g., First Nations, Métis, Inuit)	18	11.32
Black (e.g., African American, African)	4	2.52
East Asian (e.g., Chinese, Korean, Japanese, Taiwanese, Mongolian)	7	4.40
Latinx/Hispanic (e.g., Mexican, Argentinian, Cuban)	5	3.14
South Asian (e.g., Indian from India or Uganda, Pakistani, Punjabi, Tamil)	2	1.26
West Asian or North African (e.g., Armenian, Moroccan)	1	0.63
Middle Eastern (e.g., Syrian, Egyptian, Iranian, Saudi Arabian)	3	1.89
Other	3	1.89
Prefer not to answer	5	3.14
**Level of education**		
Bachelor's degree	47	29.56
College diploma	30	18.87
Master's degree	28	17.61
Some post-secondary education	23	14.47
Doctoral degree	12	7.55
Some high school	6	3.77
Less than high school	3	1.89

Four participants did not consent to sharing their demographic information.

^*^Gender identity was recorded as Male, Female, or Prefer not to answer in demographic data collection. To differentiate between gender identity and sex assigned at birth, gender categories have been relabeled as Man and Woman.

### 3.2 Categories identified

**Figure 3:**
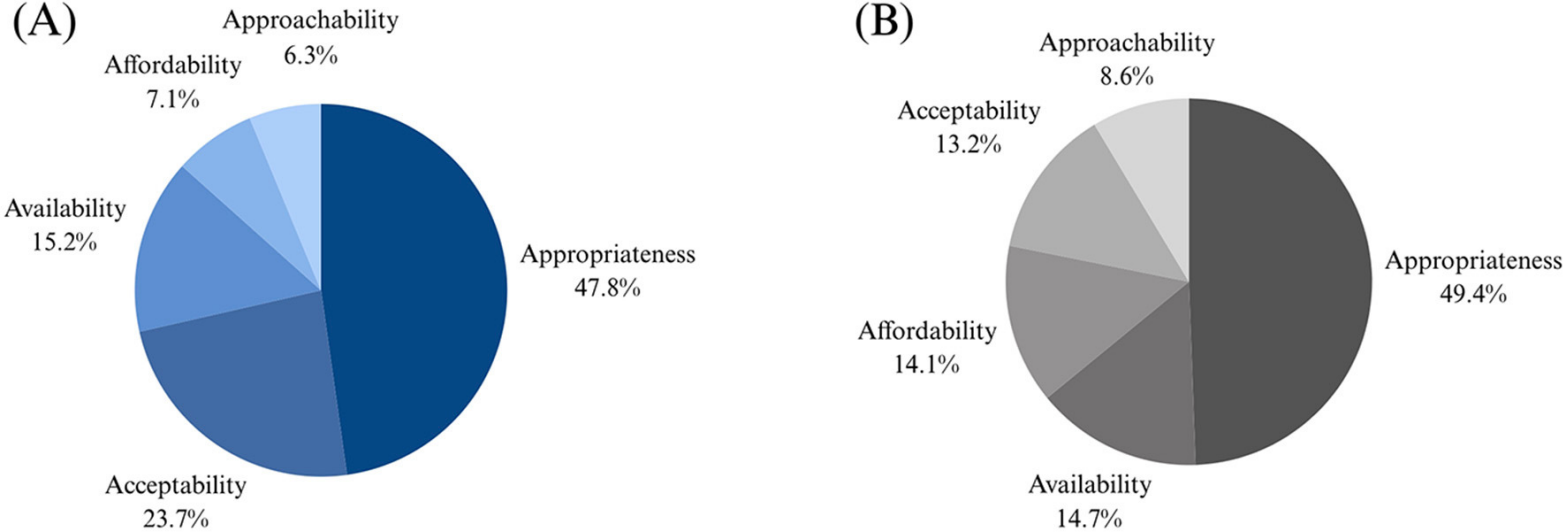
Results from preliminary coding. This figure demonstrates the distribution of coded data for the present study into the five dimensions of accessibility from the Levesque et al. (46) conceptual framework of access to healthcare. **(A)** Represents the distribution of data from facilitators, while **(B)** reflects the distribution of barriers.

There was strong inter-rater agreement (90%) between independent coders. For both barriers and facilitators to housing and healthcare, *Appropriateness & Ability to Engage* was the largest category, encompassing almost 50% of the data. The full distribution of coded data into the Levesque et al.'s (46) dimensions of accessibility is presented in Figure 3. A total of 10 distinct categories arose, five of which described barriers and five described facilitators. The overarching barriers included *Stigma, Insufficient Investment, Siloed Systems, Generalized Approaches to Housing*, and *Policies that do not Support Complex Needs*, while facilitators included *Increasing Discourse on the Intersections of ABI, MHSU, and Homelessness, Government Commitment to Systemic Change, Collaboration Across Organizations, Community-Based Services*, and *Supportive Housing Models*. The content of these categories is discussed in detail below, with examples from data and supplementary quotes provided in Tables 2, 3.

**Table 2:** Barriers to housing and healthcare: categories with representative data and quotes.

**Categories**	**Sub-categories**	**Examples from data and representative quotes**
1) Stigma	Stigma toward ABI, MHSU, and homelessness	1(a) Stigma creates barriers 1(b) Systems are/can be traumatizing “Equity in care doesn't exist because of the stigma.”
2) Insufficient investment	Disability and income assistance does not reflect the cost of living Limited and temporary funding contributing to housing instability Private and public funding discrepancies	2(a) Income for people with disabilities doesn't cover basic housing 2(b) Disability checks [are] not matching [the] costs of living 2(c) Not enough transitional housing 2(d) Inadequately resourced supportive housing to stably support people with brain injury 2(e) People are put into supported housing for only short periods of time 2(f) Discrepancy between reality and private market rates for housing “In an emergency, there is temporary funding for housing, why can't it be permanent?”
3) Siloed systems	Limitations on the comprehensiveness of support provided Difficulty navigating through the healthcare and housing systems Fragmentation in care coordination	3(a) Silos between addiction, mental health, and brain injury 3(b) Waitlists for medical/mental health supports and services 3(c) Medical forms are expensive and people with brain injuries may have difficulties with filling them out 3(d) If people in ER don't have a home to go to, they are released onto the streets “The gaps in our systems are too wide, models are not thought out well enough, and organizations need to come together.”
4) Generalized approaches to housing	“One-size-fits-all” housing to accommodate a continuum of needs Safety concerns within housing	4(a) People have unique needs along their journey: can't have one-size-fits-all housing 4(b) Need for a more realistic housing continuum based on recovery and active addiction 4(c) Lack of safe/secure item/personal belonging storage 4(d) Housing that is provided isn't safe 4(e) Housing that proves to be higher risk than being homeless “You can't put everyone with a brain injury in the same box.”
5) Policies that do not support complex needs	Stigmatizing and discriminatory policies Unstandardized training and service delivery models Meeting criteria for services	5(a) Police involvement - criminalization of homelessness/addiction 5(b) Overly restrictive rules within housing 5(c) Evictions due to BI i.e. noise, lack of social skills, too low rent payments 5(d) A lack of system in government and different levels of service 5(e) Training of staff is lacking 5(f) Fear of screening for TBI - creates barriers 5(g) Treatment according to diagnosis rather than needs “There is a disconnect between front line staff and policy makers.”

**Table 3:** Facilitators to housing and healthcare: categories with representative data and quotes.

**Categories**	**Sub-categories**	**Examples from data and representative quotes**
1) Increasing discourse on the intersections of ABI, MHSU, and homelessness	Growing public awareness Research to understand intersecting needs Advocacy	6(a) Shift in focus of research from sports to social and public health 6(b) Advocacy work, getting attention of ministry “It's taken a long time to get people on board to recognize that these populations co-exist, but it is game changing to finally have something to support some of these populations.”
2) Government commitment to systemic change	Support for informed changes Some resource allocation to new programs and ABI associations	7(a) All levels of government are starting to listen 7(b) Individual communities are building housing in their area 7(c) Lived experience & advocates at the center of the conversation 7(d) Survivors voices/experiences are being platformed “Bill C-277 is bringing awareness. It's bringing more people together.”
3) Collaboration across organizations	Integrating resources to better meet individual needs Conversations to promote coordination	8(a) Increased conversation and awareness on collaboration between organizations 8(b) One door to direct toward different resources (housing, rehabilitation etc.) “When organizations advertise their services… and network in the community… they are more easily accessible and known to everyone… This allows for the filling of gaps.”
4) Community-based services	Overdose prevention sites Brain injury associations: Peer support and outreach services	9(a) Overdose prevention sites are working 9(b) Outreach is working 9(c) Peer support groups/family support groups 9(d) Advocacy through local brain injury agencies for housing helps 9(e) Connecting with a professional assists with navigating the health care system “Community supports like [name of brain injury association] fill gaps.”
5) Supportive housing models	Complex care & transitional housing Wrap-around support in housing	10(a) Complex care programs - different types and progression (Village of Langley) 10(b) Recognition that additional supports (besides the housing) are needed 10(c) Having supports in supportive housing “[Offering] different types of complex housing allows people to move through the spectrum of housing.”

### 3.3 Barriers to housing and healthcare

#### 3.3.1 Stigma

Stigma emerged as a foundational barrier impacting multiple levels of service access and delivery. Participants described pervasive negative attitudes and discrimination toward individuals with ABI, MHSU, and PEH (1a). These attitudes, often grounded in fear, misunderstanding, and judgment, were seen in both public perceptions and interactions with service providers. Stigma was also described as contributing to re-traumatization within these systems (1b) which, in turn, can discourage help-seeking and erode trust in services. Participants also identified stigma as a key driver of inequities in service design and delivery, with one participant stating, “Equity in care doesn't exist because of the stigma”.

#### 3.3.2 Insufficient investment

Economic barriers, including insufficient government investment into housing and income supports, were identified as key barriers to obtaining housing and required services. Participants described how low investment has rendered “affordable” housing financially inaccessible to many survivors of ABI and PEH. Importantly, this is paralleled by an underinvestment in disability and income assistance funding, leaving individuals who rely on these supports as their only income significantly restricted in their ability to afford market housing. The shelter portion of disability and income assistance was described as inadequately aligned with current housing costs (2a). Participants further noted that the remaining funds, once housing costs were accounted for, were insufficient to meet basic needs given the rising cost of living (2b) and the need for specialized services essential to an individual's rehabilitation and recovery that are not covered by public funding (e.g., occupational therapy, psychotherapy, physiotherapy).

Participants also highlighted an underinvestment in existing transitional and supportive housing structures, contributing to a limited supply of appropriate housing options (2c). They described how these supports were often funded through limited and temporary resources, leading to instability in service availability and delivery. One participant asked, “In an emergency, there is temporary funding, why can't it be permanent?” Participants noted how this inconsistent investment (2d), often resulting in shorter-duration accommodation for individuals in need (2e), was harmful in perpetuating cycles of homelessness, particularly for survivors of ABI who require long-term, continuous support. Additionally, participants raised concerns about the disparities between private and public funding, noting that privately funded services are often financially inaccessible (2f) and that the widening gap between public and private housing markets further restricts housing opportunities.

#### 3.3.3 Siloed systems

Participants described the siloed nature of healthcare and housing services as a significant barrier to accessing appropriate supports (3a). Many participants reported difficulties navigating through the fragmented systems, facing long waitlists (3b) and challenges in getting connected to appropriate services. Technical barriers, such as complex paperwork and application processes for housing, medical services, and funding, were noted as particularly burdensome for ABI survivors who may require assistance to complete such tasks (3c). Participants also highlighted how the siloed structure of these systems contributes to fragmentations in care. For example, participants described an emphasis on acute care models with limited post-injury support for social reintegration and ongoing needs. Similarly, transitions between care (e.g., from youth to adult services, or from integrated care back to the community) were described as lacking continuity, often leaving survivors without coordinated, long-term support in place. Participants also emphasized the consequences of poor continuity and coordination of care for PEH, noting how individuals are released from hospitals without appropriate follow-up (3d).

#### 3.3.4 Generalized approaches to housing

Employing what participants referred to as a generalized or “one-size-fits-all” approach to housing solutions was recognized as a barrier to obtaining and benefiting from housing for individuals at the intersections of ABI, MHSU, and homelessness. Participants described how these generalized approaches fail to accommodate individual needs (4a), such as support with activities of daily living or physical accessibility requirements. Moreover, participants relayed the importance of recognizing the broad continuum of needs that exist within the intersections of ABI, MHSU, and homelessness (4b). This was reinforced by one participant, who noted, “You can't put everyone with a brain injury in the same box”. Some areas that participants reported are not being met by generalized housing approaches included accommodating younger individuals who require support, accommodating independent or high-functioning survivors of ABI who require minimal support, and finding solutions for PEH who do not have access to secure storage for their personal belongings (4c). Participants also raised safety concerns within generalized housing approaches (4d), describing inadequate structures to ensure the safety of vulnerable people within housing services. More specifically, participants noted the limited safe housing options for women, especially for those escaping violence. Some participants shared that, in their opinion, the safety risks associated with residing in certain generalized housing environments outweighed those of being unhoused (4e).

#### 3.3.5 Policies that do not support complex needs

Current policy frameworks were described as insufficient in meeting the reality of needs among service users and service providers to guide appropriate support for concurrent ABI, MHSU, and homelessness. This discrepancy was highlighted by one participant, who stated, “There is a disconnect between front line staff and policy makers”. Participants identified certain policies relating to the criminalization of homelessness and MHSU (5a) alongside restrictive housing policies (5b) that they described contribute to stigmatizing and discriminatory practices. Specifically, participants described how certain housing policies were biased against survivors of ABI, given that individuals' ability to adhere to many housing rules (e.g., noise, rent payment, social conduct, and maintenance of living space) may relate directly to ABI symptomatology and areas where support or accommodations are needed (5c). Additionally, participants reported insufficient frameworks to standardize approaches across organizations and communities (5d). Participants shared how the enforcement of bylaws and delivery of care varied widely, which has had downstream effects on service users' experiences in accessing care. Moreover, participants noted how current policies are insufficient in providing guidelines for responding to complex needs and outlining best practices for support services and housing. The need for standardization was particularly relevant to education for service providers and healthcare professionals (5e). Participants described an inconsistency in the recognition and diagnosis of ABI, as well as a reluctance to screen for ABI (5f).

Given the insufficient training and resources for healthcare providers to adequately diagnose ABI, participants raised concerns about policies that require diagnoses for eligibility to access funding and support services (5g), noting that when support is contingent upon diagnoses and systems cannot provide screening, these services remain inaccessible to individuals who would otherwise benefit from them. Additionally, participants described discrepancies in the kinds of support offered based on the cause of ABI (e.g., differences in funding for motor vehicle accidents compared to other sources of ABI, such as intimate partner violence or overdose survival). Meeting criteria for diagnoses and support services was noted by participants as particularly challenging among PEH, who may have limited access to supporting information such as personal identification, an address, and medical documentation.

### 3.4 Facilitators to housing and healthcare

#### 3.4.1 Increasing discourse on the intersections of ABI, MHSU, and homelessness

While stigma remains a significant barrier, participants articulated an encouraging shift toward greater awareness among the general public that ABI, MHSU, and homelessness are interrelated challenges. This idea was emphasized though one participant's statement, “It's taken a long time to get people on board to recognize that these populations co-exist, but it is game changing to finally have something to support some of these populations”. Participants also expressed how there has been a shift within the ABI research community toward public health, seeking to understand the needs within these populations to inform evidence-based supports (6a). Participants shared how this shift in research focus, combined with an increasing public awareness, has helped to advocate for change. Credited to this advocacy, participants reported obtaining recognition at the systemic level (6b), receiving acknowledgment of this pressing public health concern from all levels of government.

#### 3.4.2 Government commitment to systemic change

Participants relayed the importance of government involvement as a facilitating factor to systemic and policy change. Several participants reported that all levels of government are beginning to acknowledge the intersections of ABI, MHSU, and homelessness and are motivated to support change that will address barriers to care (7a). Some participants reported that municipal governments are beginning to take initiative in developing new housing that will better serve individuals among these intersections within their own communities (7b). Similarly, participants discussed ongoing efforts to respond to increasing needs for transition housing and funding allocations to ABI services and low-barrier programs.

Participants also noted the recent involvement of individuals with lived experience of these intersections in government conversations for policy and service development (7c). They shared how advocates are being heard at the systems-level and survivors' stories are being acknowledged and shared (7d). Additionally, participants described support from government representatives for a National Strategy on Brain Injury that would help to address several barriers to and gaps within current systems. Moreover, participants shared insights to how the promotion of a National Strategy on Brain Injury within Canada has helped to increase awareness. In discussions about a National Strategy on Brain Injury, one participant shared, “Bill C-277 is bringing awareness. It's bringing more people together.”

#### 3.4.3 Collaboration across organizations

In an effort to bridge gaps between siloed systems, participants described the importance of collaboration between organizations as a facilitator to improving housing and healthcare accessibility (8a). Participants described how integrating information about services has helped to tailor resources and recommendations to individual needs and reduce the need for systems navigation (8b). Likewise, participants noted how conversations between organizations have helped to both promote awareness of which services are available and how different stakeholder groups can facilitate connections and collaboration between their respective agencies and the services they provide. On this topic, one participant stated, “When organizations advertise their services… and network in the community… they are more easily accessible and known to everyone… This allows for the filling of gaps”.

#### 3.4.4 Community-based services

Community-based services, including local brain injury associations and overdose prevention sites, were recognized as providing critical support (9a). Among other services offered through brain injury associations, participants described the effectiveness of outreach programs (9b) as well as peer and family support groups (9c). Some participants described housing outreach programs offered through these organizations, which help to reduce technical application barriers to seeking and obtaining housing. This advocacy and support with systems navigation was reported as effective for promoting access to housing (9d) and healthcare (9e). Participants also shared their appreciation for non-profits and community-based organizations recognizing the needs within their communities and working within their capacities to address them, with one participant saying, “Community supports like [name of brain injury association] fill gaps.”

#### 3.4.5 Supportive housing models

Lastly, participants described the effectiveness of supportive housing models, including complex care and transitional housing, in meeting individual needs for people experiencing the intersections of ABI, MHSU, and homelessness. Participants mentioned the importance of offering a progression of housing options that reflect varying levels of independence and support (10a), such as moving from hospital discharge into transitional or semi-independent living environments. One participant highlighted the need for this progression in saying, “[Offering] different types of complex housing allows people to move through the spectrum of housing”. These housing models were commended by participants for their flexibility, especially for individuals whose needs may not be reflected in conventional housing expectations. Participants also emphasized the importance of wrap-around supports embedded within these housing models (10b, c). Having assistance with activities of daily living, such as cooking, physical care, and medication management, was reported as a critical component to meeting individual needs and promoting positive outcomes within housing environments.

## 4 Discussion

This study aimed to identify the barriers and facilitators to housing and healthcare that exist for PEH with concurrent ABI and MHSU. Additionally, this research aimed to contribute to the overarching goal of the BC Consensus on Brain Injury research project by supporting the development of a consensus that reflects the priorities and solutions to best serve individuals experiencing these intersections. Through a collaborative, interdisciplinary approach, five key barriers and five facilitators were identified. These findings contribute to a growing body of literature that highlights the urgent need for system-level change, and they provide actionable directions for policy, service delivery, and future research.

The barriers and facilitators identified in this study speak to the difficulty of navigating between systems and the need for integrated approaches. Consistent with previous literature (2, 37), this study identifies the fragmentation between housing, healthcare, mental health, substance use, and ABI supports to be a significant barrier in accessing support. Past research emphasizes the lack of coordination between these systems as contributing to gaps in knowledge among service providers and accessibility challenges for service users (2, 14). Moreover, the siloed nature of these systems can perpetuate delayed access to care due to difficulty identifying resources and long wait times (14). Disconnected systems and their effects on service comprehensiveness was especially pronounced for PEH, given their experiences being discharged from care back to the streets. Previous studies have emphasized this significant gap in care delivery, recommending that policy changes are developed in partnership with healthcare and housing representatives and people with lived experience of homelessness (56).

Participants relayed the effectiveness of collaboration between organizations, resource integration, and outreach services offered through community supports in helping to dismantle the barriers created by siloed systems. These findings echo previous work, which has recommended reducing the need for survivors of ABI to independently coordinate access to services, particularly when they are dealing with concurrent MHSU challenges (2). Opportunities to implement this support could include a system navigation support worker (37), community partnerships across multiple service delivery sectors (e.g., harm reduction, mental health, housing, etc.) (2, 25), integrated or co-located services (2, 14), or sharing information about available resources in one centralized location (14). Improved coordination, collaboration, and conversation between stakeholders and services has also been endorsed as an essential component to improving post-discharge outcomes for PEH (56).

Participants also identified several barriers that exist within healthcare service provision. Upon accessing healthcare services, participants described the insufficient screening and diagnosis of ABI as a barrier to receiving adequate support, both financial and rehabilitative. This gap in care was largely attributed to insufficient policies to prioritize standardized, comprehensive education for service providers to identify and diagnose ABI, particularly in the presence of concurrent MHSU challenges. These findings are in accordance with previous studies, which have emphasized the need for service provider education and improved ABI screening and diagnosis (2, 14, 16, 37, 41). Specifically, it is important for physicians to understand the complexities and ongoing vulnerabilities of ABI survivors to accommodate long-term supports (16) and to provide a timely diagnosis to facilitate early access to support (14). Screening at common points of care for PEH, such as primary care centers, MHSU services or within shelters, has been highlighted as a promising practice for connecting PEH with the services they need (16, 33). While doing so may detect ABIs that have been missed in previous evaluations, lifetime screening for ABIs is still not a common practice within these settings. In addition to training on ABI recognition, providing training for essential skills such as harm reduction, crisis support, and de-escalation to all service providers has been recommended to address knowledge gaps and improve service delivery to survivors of ABI with concurrent MHSU challenges (37). Interdisciplinary research that involves people with lived experience, such as the present study, may serve to support the development of these educational resources, given that systematic reviews have advocated for formalized training about ABI, MHSU, and homelessness that incorporates the perspectives of both service providers and service users (2). Given the inconsistencies in ABI screening and diagnosis, particularly in PEH and MHSU populations where mental and physical health conditions may overshadow symptoms of ABI, participants also highlighted that diagnosis-contingent services are a barrier for individuals navigating concurrent challenges. Previous research has also highlighted this barrier (14, 16, 37), with some recommendations suggesting lowered thresholds for referrals to additional support among this population (16).

Regarding housing, participants identified an insufficient investment in disability assistance and supportive housing models as barriers to obtaining appropriate housing, while generalized approaches to housing were reported as unsafe and failing to accommodate individual needs. In their review of the critical characteristics of housing for survivors of ABI with concurrent MHSU challenges, Estrella and colleagues (37) identified similar affordability and accommodation barriers. They noted that survivors had few options for affordable housing, particularly when they had limited income or were unemployed. While some federal funding has been allocated to develop transitional or affordable housing models, including housing with wrap-around MHSU care for veterans experiencing homelessness (57), further advocacy is needed to secure comparable funding and support for non-veteran populations. Specifically, there is a critical need to expand these initiatives to address neurocognitive impairment following ABI as part of integrated wrap-around services. Among other critical characteristics for housing stability and satisfaction, researchers identified felt safety and wrap-around support as being important for survivors of ABI with concurrent MHSU challenges–two integral areas that generalized approaches to housing do not reflect. Insufficient funding as it relates to healthcare services was also identified by Grewal and colleagues (14), contributing to unpredictable availability of services. In contrast to generalized housing approaches, participants shared their support for complex care and transitional housing models that integrate a variety of support, including support with activities of daily living, into housing environments. These supportive housing models align with findings and recommendations from previous literature, including the need for holistic approaches to housing and rehabilitation (41), integrated services in housing (37), and support with life skills development (14).

Lastly, participants reported stigma as a pervasive barrier to equitable healthcare and housing opportunities. This was evident among other studies, which highlighted how ABI survivors with concurrent MHSU challenges faced disadvantages in the housing rental market due to discrimination from housing providers and landlords (37). Participants also identified how certain housing policies may be discriminatory toward ABI survivors, given that their content reflects common challenges that often directly relate to ABI symptomatology. For example, policies that relate to standards for cleanliness in living spaces may provide a basis for subjective and discriminatory practices against survivors of ABI who require additional support to complete activities such as cleaning and maintaining their space. These policies highlight critical areas where considerations and revisions are needed to support individuals in obtaining and maintaining housing.

Discriminatory practices are evident in healthcare settings as well, where individuals may receive substandard care due to biases (14). Importantly, perceived stigma, both in healthcare and housing contexts, may prevent an individual from sharing their needs or disclosing an ABI, leading them further away from obtaining resources and support (14, 32). While stigma remains a significant barrier that needs to be addressed at all levels of service design and delivery, participants shared an encouraging shift toward increasing levels of awareness among the general public about relationships between ABI, MHSU, and homelessness. Compared to findings from Year One of the BC Consensus on Brain Injury, which indicated a lack of awareness among the general public (14), these findings highlight the growth that has come from advocacy efforts by communities, organizations, and researchers. The importance of advocacy to counteract stigmatizing beliefs and practice is also endorsed by earlier research (14). These advocacy efforts will also be crucial to addressing stigma and discriminatory practices in housing infrastructure and policy.

This study contributes an important perspective to understanding the intersectionality of ABI, MHSU, and homelessness as it relates to service utilization and provision. It directly aligns with recommendations from previous studies to engage multiple community partners as knowledge holders (e.g., landlords, service providers, individuals with lived experience) in the research process to understand these intersecting challenges holistically (37). The PAR methodology (44) underpinning the current study also provides a valuable contribution to existing literature. By involving knowledge holders in facilitated discussions, rather than individual interviews, participants were able to generate collaborative insights to the challenges and benefits of existing housing and healthcare models that reflect their unique knowledge and lived experience. This facilitated form of knowledge sharing and idea generation also allowed for all participants to be heard, which was important for individuals with lived experience of ABI or homelessness and to understand the needs of service providers to improve care within these intersections. Importantly, this study highlights areas of service design and delivery that are working well. While it is crucial to acknowledge the limitations of existing services, it is also essential to identify and leverage approaches that are effectively supporting people within this population, especially given the pressing nature of these public health concerns and the cycles of vulnerability many individuals face.

### 4.1 Implications for research and clinical practice

This research begins to explore the challenges associated with concurrent ABI and MHSU in the context of homelessness. However, there remain several opportunities for future research to improve outcomes for individuals affected by these intersections. A recent study by Kennedy and colleagues (45) identified research priorities to address the intersections of ABI and MHSU. While the present study begins to respond to Kennedy et al.'s number one priority of understanding the differences between homeless or marginally housed people and people with stable housing in their experiences of ABI and MHSU, additional research is still needed to explore subsequent priorities including understanding the prevalence of non-fatal opioid overdose-related hypoxic/anoxic brain injury and effective supports for survivors. Related, future research could address stakeholder-generated priorities of evaluating and optimizing existing interventions for immediate implementation, developing specialized interventions and diagnostic techniques, and collecting meaningful data to better understand the impacts and intersections between ABI, MHSU, and homelessness (33). Additionally, while it was not the goal of the current study to explore in-depth personalized accounts of experiences within these intersections, future research could explore this lens and contribute informative work that solely highlights barriers and facilitators from the perspective of those with lived experience.

At the clinical level, improvements can be made to ABI screening and the assessment of needs among this population to facilitate access to appropriate support. The Vulnerability Assessment Tool (58), a method to evaluate a person's vulnerability to continued housing instability and homelessness, can be used to identify individuals who would benefit from services such as complex care and case management. Similarly, the Brain Injury Screening Index (59) is one method of screening for ABI, which could support healthcare providers in standardizing their approaches and increase survivors' likelihoods of getting connected to resources. Therefore, systematic screening for lifetime ABIs should be implemented during intake for MHSU and PEH-specific services, given the high burden of ABI in these populations (15, 16, 24). Additionally, screening tools could be implemented by first responders, including following overdose events, to connect individuals to services and resources they may otherwise not encounter. Other stakeholder-driven recommendations to advance clinical practice in serving PEH with ABI and MHSU include providing affordable and accessible supportive housing, enhancing resources to service providers, designing needs-based services that promote quality of life, improving communication and collaboration between service providers, adopting long-term integrated approaches to care, and reducing stigma and discrimination through standardized education (33).

## 5 Limitations

This study has some notable limitations. While this project aimed to capture a wide range of experiences and insights, the recruitment process was initiated through organizations. Although service providers within these organizations extended the invitation to individuals who were impacted in some way by the intersections of ABI, MHSU, and homelessness, this approach may have not reached some of the most marginalized individuals who are in need of support but are not connected with any services in their community. Similarly, there was an intentional inclusion of equity-deserving groups (e.g., Indigenous people, members of the 2SLGBTQIA+ community) who are disproportionately impacted by ABI, MHSU, and homelessness (50–54). However, because of these impacts, the study may not have been representative of the full continuum of needs among this population. As previously mentioned, individuals with lived experience who did participate received compensation in accordance with the BC Center for Disease Control Peer Payment Standards (55), which helped to minimize financial barriers to participation (e.g., compensation for childcare coverage, missed work, or travel costs). Had this support not been provided, there would have been further limitations on the representation and meaningful inclusion of individuals with lived experience. Additionally, due to the provincial scope of the research questions, the barriers and facilitators identified may not reflect the policies or priorities of other provinces and territories within Canada. Future research could examine the generalizability of these findings to other regions of Canada and internationally. Lastly, while quotes were not attributed to individual participants, this was reflective of the intentionally broad scope of the research, which sought to build a consensus that incorporated multiple diverse perspectives coherently rather than focus on individual narratives.

## 6 Conclusion

The purpose of this study was to understand the barriers and facilitators to housing and healthcare for PEH with concurrent ABI and MHSU. Future efforts should continue to build on these findings by advancing inclusive, equitable policy changes, reducing stigma through awareness and education campaigns, improving cross-sector collaboration and training, and increasing funding to integrated, low-barrier housing and healthcare services that are tailored to support the unique needs of individuals within this population.

## Data Availability

The datasets presented in this article are not readily available because of ethical considerations. Participants provided informed consent with the understanding that their data would not be made publicly available within research publications. Requests to access the datasets should be directed to Dr. Mauricio A. Garcia-Barrera, mgarcia@uvic.ca.
